# Keeping Stallions in Groups—Species-Appropriate or Relevant to Animal Welfare?

**DOI:** 10.3390/ani11051317

**Published:** 2021-05-04

**Authors:** Heidrun Gehlen, Katrin Krumbach, Christa Thöne-Reineke

**Affiliations:** 1 Equine Clinic, Veterinary Department, Freie Universitaet Berlin, 14163 Berlin, Germany; pferdeklinik@vetmed.fu-berlin.de; 2 Animal Behavior and Laboratory Animal Science, Institute for Animal Welfare, Veterinary Department, Freie Universitaet Berlin, 14163 Berlin, Germany; Christa.Thoene-Reineke@fu-berlin.de

**Keywords:** stallion husbandry, horse husbandry, group housing, diseases based on husbandry

## Abstract

**Simple Summary:**

The stress of isolation from the individual husbandry of stallions can result in behavioral problems, aggression, and diseases of the respiratory, nutritional, and musculoskeletal systems. Several examples from practice show that the keeping of stallions in groups is possible in principle. It only poses a risk for injuries if the necessary requirements for this type of husbandry are not taken into account. If the size and design of the exercise area/pasture, the group constellations, and the characters of the stallions are considered, keeping stallions in groups represents the most species-appropriate form of husbandry for them. This takes into account animal welfare aspects and complies best with the requirements of modern horse husbandry. However, the integration of the stallion into an existing group should only be carried out by qualified, experienced horse owners, who must proceed professionally and step by step. Consequently, stress, disadvantages, and the potential for injury can be reduced to a minimum or, ideally, avoided altogether, and the wellbeing and mental and physical health can be supported in the best possible way.

**Abstract:**

This literature review was aimed at analyzing whether stallion husbandry in groups is possible and desirable or poses risks. This was determined on the basis of different studies in order to be able to give practical recommendations from the viewpoint of animal welfare. Consequently, 50 different sources were analyzed, as well as observations of an experiment of the Swiss National Stud on the subject of change from single-stallion to group husbandry and its influence on animal welfare. The results revealed that stallion husbandry in groups is possible but still rarely practiced. It was found that 6% of stallions in 2003, more than 11% in 2012, and nearly 23% of the stallions in 2015 were kept in groups. Furthermore, studies showed that the still widespread individual husbandry of stallions has a negative impact on psyche and body health. Almost half of all stallions showed undesirable patterns of behavior, mostly stallions in individual housing. In addition, many of the latter stallions had problems with their respiratory, digestive, and musculoskeletal systems, which improved when the husbandry conditions of the horses were changed, with the exception of the problems with the digestive system. Conversion into group husbandry is possible, as revealed by an experiment by the Swiss National Stud with a socialization of active breeding stallions outside the breeding season. Therefore, the widespread fear of serious injuries for stallions housed in groups was refuted and the aggressive behavior of the stallions decreased rapidly. Success rates for group husbandry are influenced by the individual character of the stallion, previous experience of the stallion, changes in the group, qualification and management of the farm, and organization of the group housing and husbandry system. This enables species-appropriate husbandry in groups while also considering animal welfare without stress, disadvantages, and serious injuries for stallions.

## 1. Introduction

The demand for keeping horses in groups is growing due to an increased social awareness of animal welfare [1]. Owners are also aware of the importance of keeping horses in a species-appropriate manner for their wellbeing by keeping them as close to nature as possible, i.e., living in a herd and the natural environment. Horses are very social animals that live in groups in the wild, and husbandry methods in recent years have increasingly aimed at species-appropriate management in groups [2,3]. By contrast, however, stallions are still kept in the traditional way [4,5,6] and, according to a survey from a province in Germany (Baden-Württemberg), in which 20 farms keeping breeding stallions were examined, 74% of the stallions are still kept individually in indoor stalls [7].

Problems occur due to this solitary confinement regarding the behavior and handling of the stallion [8], thereby influencing its wellbeing [5] and health [9]. The guidelines for the assessment of horse husbandry under animal welfare aspects (Federal Ministry of Food, Agriculture, and Consumer Protection (BMELV) and the German Animal Welfare Act (TSchG) [1]) state that basically all horses are suitable for group husbandry regardless of their age, breed, sex, and type of use [10].

Furthermore, the ethical principles require that the husbandry of a horse should take into account its natural needs [11]. This is still opposed by the outdated attitude that stallions have always been kept singly and the fear of injury when kept in a group due to the assumption of the dangerous nature of stallions among many stud owners [12]. However, visual, auditory, and olfactory contact between animals is a minimum requirement in horse husbandry [10]. Nevertheless, the issue of keeping stallions in a species-appropriate manner is currently of utmost relevance, as more and more stallions are being kept privately [13], for example, for baroque riding [6]. Optimization of the type of husbandry is, therefore, the order of the day for the farms [14].

Therefore, the aim of the present work was to verify, on the basis of a literature research, whether it is possible, from an animal welfare point of view, to keep stallions in groups and, thus, to enable them to be kept in a manner appropriate to their species.

## 2. Materials and Methods

The literature search was performed using the databases of the University Library Primo of the Karlsruhe Virtual Catalog, as well as PubMed and Google Scholar on the internet, by entering keywords (stallion, stallion husbandry, species-appropriate husbandry, group husbandry, husbandry-related diseases). In addition, the Veterinary Medicine University Library of the Free University of Berlin was searched for appropriate literature. Legal texts were taken from the database “gesetze-im-internet.de”. Furthermore, studies could be found via the internet search engine Google and at FNverlag. Lastly, a literature research was conducted in documents, books, and brochures that were available on-site in the university library.

The studies were included in the literature review if they dealt with the husbandry of stallions, group husbandry, and/or husbandry-related diseases. Consequently, the title and table of contents of the publications were first examined for relevance to the present work. Afterward, the summary, conclusion, and the most essential aspects of the respective study were read in order to facilitate the answering of the underlying research question. The types of studies and documents were not considered as inclusion or exclusion criteria since only a few sources could be found. The publications were also limited to after 1990 in order to obtain as current a situation of stallion husbandry as possible. This ultimately resulted in 50 different sources for analysis.

Despite the small number of publications, a high quality could be achieved due to the use of mainly scientific texts (such as dissertations). The research was limited predominantly to German and English literature.

## 3. Results

Four German publications from the analysis of various sources reflected the repetitive and similar results particularly well. Therefore, they are presented here in a little more detail.

The study by Pollmann et al. [7], in which the housing conditions of 66 stallions, more precisely, 29 small horses (<148 cm) and 37 large horses (>148 cm), were investigated in 20 farms with breeding stallions in Baden-Württemberg, showed that only 6% of the stallions were kept in groups. However, these were only small horse stallions, the majority of which were allowed free exercise with other horses, such as mares, geldings, or young stallions, in the open air or on the pasture and, thus, with social contact.

By contrast, the study by Irrgang and Gerken [4] found that 29.5% of the stallions on 29 farms, where 78 Arabian stallions were stabled, were kept in groups. This work aimed to determine the extent to which alternative housing systems are already practiced in the keeping of Arabian stallions and whether there are differences in rearing, husbandry, management, and handling, as well as the effects of husbandry on the behavior of the stallions toward their conspecifics and humans. Their results proved that keeping stallions in groups in the most diverse combinations is possible in principle and has a positive influence on the behavior of the stallions. However, their husbandry depends largely on their rearing. In addition, 45% of the stallions, which were mostly kept in solitary confinement, showed undesirable behavior (i.e., aggressiveness, stereotypies, and self-mutilation).

A more recent study by Steiner [15] analyzed 96 farms and their housing conditions for a total of 463 stallions in Lower Saxony (province in Germany). Almost 12% of the stallions were kept in groups. These were both stallions in stud use and those not in use for breeding. Therefore, the sex ratio and the available usable area were very different in all farms. The analysis also showed that warmblood stallions were more often kept in indoor stalls than stallions of the thoroughbred and pony breeds.

The husbandry of adult stallions in Bavaria was reviewed by Zilow [6], according to compliance with the guidelines for the evaluation of horse husbandry under animal welfare aspects [10], on 67 farms with a total of 101 stallions. The effects of different management and husbandry conditions on the behavior of the stallions were investigated. Almost 23% of the stallions were kept in groups of different size and composition. The study showed that group housing of stallions including studs is possible in principle and has a positive effect on behavior. By contrast, almost half of the stallions that were mostly kept in single housing showed undesirable behavior (such as aggressiveness, stall or fence running, gnawing of wood and feeding through, and excessive tail rubbing and tongue play while riding). Breeding was defined here as a decisive influencing factor.

### 3.1. Results from the Trial on the Group Housing of Stallions at the Swiss National Stud

**Figure 1:**
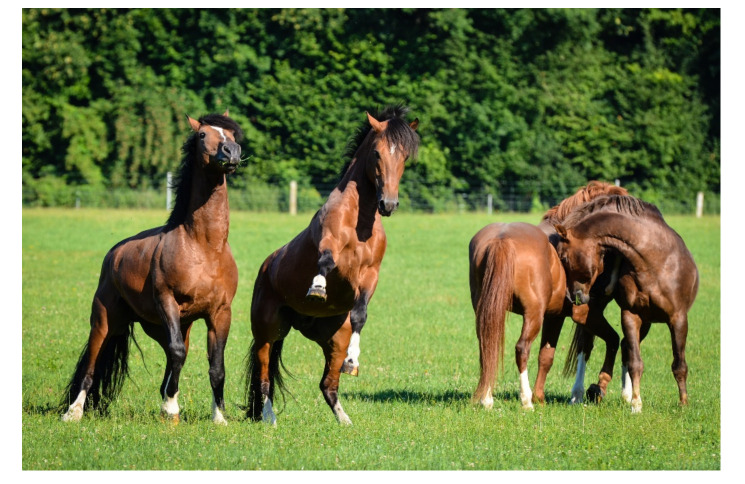
A group of stallions enjoying social contact on pasture (Agroscope, Schweizer Nationalgestüt, Switzerland).

Briefer Freymond et al. [5] investigated whether group housing of stallions is possible by accustoming active breeding stallions to being kept in a group outside the breeding season over a period of 6 months. To accomplish this, increasing numbers of stallions were socialized in a group in a 4 acre pasture during the period from 2009 to 2010 (Figure 1).

**Figure 2:**
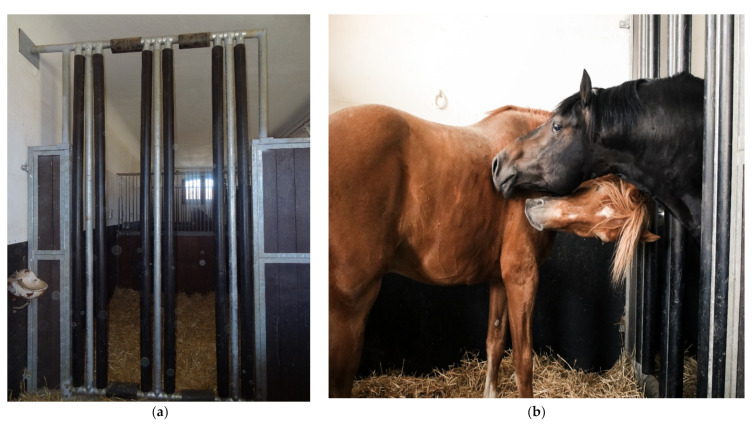
(**a**) Socialbox (Agroscope, Schweizer Nationalgestüt, Switzerland). (**b**) Socialbox with first contact of two stallions (Agroscope, Schweizer Nationalgestüt, Switzerland).

This was preceded by a 2 week acclimation period in neighboring stalls, where they were able to make contact with each other for the first time (Figure 2a,b) before being transferred to the pasture with sufficient escape routes, a sufficiently high number of feeding places, and no mare contact.

Only minor injuries were observed, as well as a decrease in aggressive behavior during the first 4 days. The stallions played with each other and established social contact in the form of mutual grooming, which had a positive effect on the mental and physical health of the animals. A generally friendlier and more relaxed attitude toward humans was observed. The authors, therefore, assumed that the keeping of stallions in groups is possible in principle and that a certain previous experience in group husbandry minimizes aggressive behavior when stallions are reintroduced into groups. They continued after each year to put stallions in groups of varied size of 5–12 stallions, and the stallions displayed more ritual than agonistic interaction. This is important because ritual interaction does not involve any risk of injury (personal communication, Briefer-Freymond).

Thus, the unanimous tenor of the studies is that keeping stallions in groups is possible per se and has a positive influence on the mental and physical health of the animals.

### 3.2. Species-Appropriate Alternatives to Group Housing of Stallions

Since unpredictable situations can always arise suddenly when stallions are kept in groups and there are also stallions with social problems that generally cannot be integrated into a group, for example, due to years of isolation, there must be alternatives to group housing for the stallions [8,16]. Group housing is also rather unsuitable for farms where horses change frequently [16]. Therefore, there are species-appropriate alternatives to keeping stallions in groups, some of which can also serve as an interim solution on the path to long-term species-appropriate keeping in groups through habituation [8].

**Figure 3:**
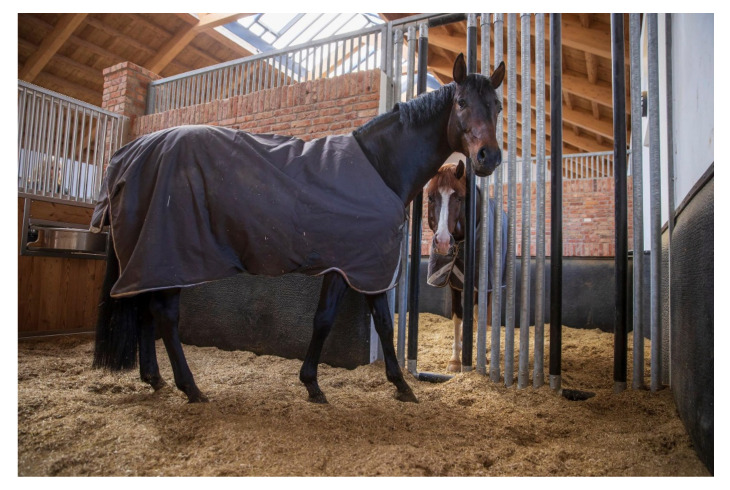
Socialbox. Gut Schönweide, Germany (Breeding Stallions Follow Him’s Schönweide und Sky).

Because a gelding is usually unproblematic for the stallion, an alternative would be to keep the stallion alone in the box overnight and with a conspecific in a shared run during the day [16]. In this way, the stallion is not kept alone in the box all the time but is allowed to run out in the pasture for several hours every day with a conspecific, thus becoming a species-appropriate alternative for the stallion. It is very rare that this is not possible and that the stallion is better off left alone [16]. However, if this is the case, at least visual, auditory, and olfactory contact between the stallions should be possible [10]. Boxes with the possibility of physical contact and withdrawal, in which the stallions stand next to each other, would be suitable for this purpose (Figure 3) [17].

**Figure 4:**
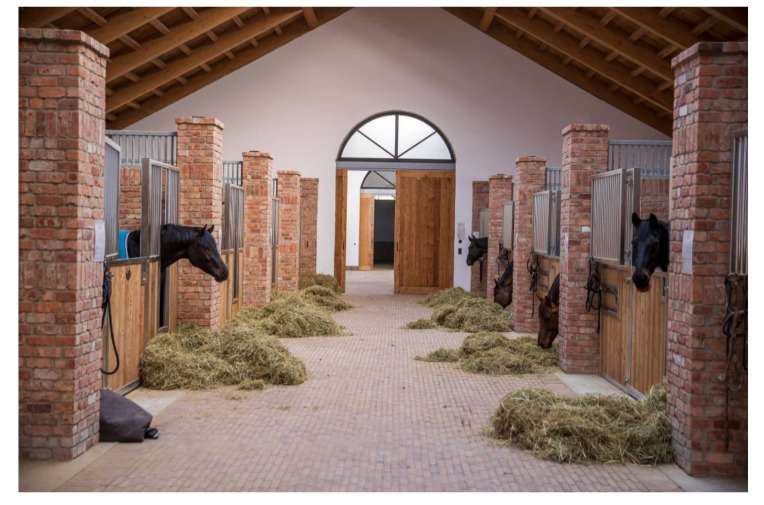
Social stall. Gut Schönweide, Germany.

There are individual stalls with special stall partitions that facilitate this (‘social stalls’). It was found that typical stallion behavior, such as displaying, was increased only in the first 2 hours after stabling in the social stalls. In addition, the opportunity for extended visual contact (Figure 4), for example, when feeding together, and for physical contact was taken advantage of (Figure 3) [18].

Another option is to keep the stallions separated in paddock boxes, ideally with adjacent pasture, as this allows social contact to be maintained across the fence. However, if the stallions are particularly incompatible, the adjacent paddock and pasture should be kept free.

An open stable is another alternative to keeping stallions in groups. In this case, an open stall with several indoor areas can be restructured to create a separate independent unit for the stallion. The separated indoor area can allow visual (Figure 4) and head contact, and the adjoining single paddock can be right next to the group paddock and enable contact or leave a few meters of free space. Similarly, an adjoining individual pasture with a few feet of clearance from the group pasture is a good idea. If the open stall offers only one indoor space, an outdoor stall can be connected near the open stall so that similar divisions and spaces can be created here [13].

If the stallion is to be kept in the pasture and neither group husbandry nor physical contact with conspecifics is suitable for it, stallions can be kept individually in the pasture with a shelter, always leaving a pasture free between them [13].

A final possible alternative to group husbandry of stallions, which was formerly used for keeping breeding stallions at stud farms, especially in British facilities, consists of buildings with indoor stalls enabling a low degree of contact, as well as attached individual paddocks with space between them [13].

Lastly, it should always be noted that castration should also be considered for animal welfare reasons for stallions that are not used for breeding and cannot be integrated into a species-appropriate husbandry (Deutscher Tierschutzbund n.d.).

## 4. Discussion

The results of this literature review facilitate the answering of the underlying questions well and in detail. It is indicated uniformly that the group husbandry of stallions is possible in principle and has already been practiced, even if so far only sporadically, in increasing instances in Germany and Switzerland. The study of Pollmann et al. already argued in 2003 for the possibility of keeping stallions in different group constellations. In 2020, Pinto and Hirata found out that some feral horse populations form multi-male groups independent of the size of the stallions [2,19]. However, not only stallions of small horse breeds are currently kept in groups. In 2008, Briefer-Ferymond integrated four stallions as a pilot phase at the Agroscope (Schweizer Nationalgestüt, Switzerland), and there was also a warmblood horse in the group. For their point of view, it is not a question of size (personal communication, Briefer Freymond). The idea of many stallion owners that pony breeds are especially more compatible and less aggressive than large horses may have had an influence here. Schmelzer (1998) [8] also assumes that the nature of ponies is different from that of large horses and that pony stallions have been commonly kept in groups for much longer. By contrast, Irrgang and Gerken recorded in 2010 that almost one-third of all Arabians are kept in groups [4].

The authors pointed out that Arabians are kept in groups at a much younger age. Steiner [15], however, pointed out that this is traditionally done only in the summer months but not in winter. Here, the author continued by stating that they are also housed alone in individual stalls because of their assumed sensitivity. Winther Christensen et al. [20] studied social behavior in domesticated groups of 2 year old stallions in comparison to a mixed age group of Przewalski stallions. They found out that social interactions and agonistic behaviors were very similar [20]. The nondomestic relatives showed even more social grooming than the homogeneous domestic group [20]. In principle, it must also be possible to keep stallions of warmblood horse breeds in groups. Steiner [15] determined that just under 12% of stallions of various breeds are kept in groups in Lower Saxony. Nevertheless, Zilow [6] also stated that the keeping of stallions of different breeds is practiced in Bavaria for just under 23% of cases.

Although the prevailing assumption that stallions cannot be kept in groups may seem antiquated, it is, nevertheless, in the course of the implementation of animal welfare, linked to certain preconditions. Thus, group husbandry can only be successful if the stress and discrimination of individual horses are avoided as much as possible, and it must be based on the premise of preventing injuries. The individuality of the respective stallion and that of the other group members, as well as the conditions on the respective farm, must be considered. This includes, above all, sufficient space. This varies particularly strongly in practice [15]. Different amounts of space are required depending on the group constellation and the varying need for individual distance of each stallion. Nevertheless, the design of the space available is also important. Dividing the space into functional areas prevents a concentration of horses and facilitates simultaneous care of all horses, which also minimizes the risk of injury. Dead-end situations must be avoided, and retreats for lower-ranking horses must be available [21]. A sufficient number of separate feeding and drinking places, as well as lying and resting areas, must be available in order to avoid stress and injuries [12,21]. This represents the needs of all horses regardless of their breed [10]. However, the individuality of stallions should not be disregarded. Not every stallion is suitable for group housing. Thus, social shortcomings and a high level of aggression have the effect of preventing integration [8]. By contrast, current breeding use is by no means an obstacle to group housing, as studies by Pollmann et al. [7], Irrgang and Gerken [4], Zilow [6], and Steiner [15] have shown. Lastly, this was also confirmed by the trial at the Swiss National Stud, where breeding stallions could be successfully integrated into a group [5], while no serious injuries could be detected. In addition, the aggression of the horses decreased significantly within 4 days. In principle, the authors assumed that an integration into a group is possible, since it is known from observations of wild herds that stallions there also live in the group, regardless of whether they are currently breeding or not. Furthermore, the researchers found that the individual age of the stallion is not decisive for the success of integration. Again, this result is not surprising when compared to wild horse herds.

Stallions, to which the opportunities for social interactions are given, may even increase reproductive efficiency [22]. It could be beneficial if grouping took place outside the breeding season and mare contact was avoided during the grouping time [5].

Much more crucial, however, is the expertise of a farm’s manager and management [21]. This must take the social structure and the compatibility of the individual horses into account [10] and carry out the integration gradually. Farms with a low turnover, in which the social structure can be kept constant and stable, therefore, have a favorable influence [21]. Independently of the presence of a stallion, group stability prevents aggressive social interactions between members of the herd [23]. Management plays a particularly decisive role in determining whether stress arises in the group and, thus, whether injuries are promoted or avoided.

If, in spite of everything, group husbandry is not possible, work must be done to ensure that the stallion is kept as species-appropriate as possible [5]. It would be conceivable to keep the stallion with a conspecific in a common run during the day and a box at night [16]. If this is also not possible, at least visual, auditory, and olfactory contact with conspecifics should be provided [10]. Integration boxes in which the stallions stand side by side with the possibility of physical contact and withdrawal are also suitable alternatives [17,24]. Paddock boxes with social contact would, nevertheless, be a species-appropriate accommodation option [25]. Alternatively, an open stall in an open stable provides a separate independent unit for the stallion with an adjacent individual paddock and pasture a few meters away from the group paddock and pasture. Incompatible stallions can also be kept at a distance from the neighboring herd with a pasture. The so-called stallion star, on the other hand, facilitates more or less possibility of contact in its indoor stalls [13].

The group husbandry of stallions can function in the most different constellations, as proven in the respective studies of Irrgang and Gerken [4], Zilow [6], and, for example, Steiner [15]. This is not surprising when observing wild horse herds and drawing comparisons from them. The work of Steiner [15] found that, when horses are kept in groups in stable systems, just over 63% of stallions are kept with one or more male horses and just under 37% of stallions are kept with mares in family groups. By contrast, when kept in groups on pasture, stallions are more often kept with mares and foals than other stallions and geldings. It is a better alternative to place high-ranking, dominant stallions together with calm geldings [16]. Rank fights and competitive behavior with subsequent injuries can then be avoided as far as possible. It is also better to avoid contact with mares [12]. Stallions can be kept with mares if the latter are in foal to the same stallion, are to be covered by the latter, or are not in estrus. They must always be separated at the onset of estrus [16]. However, one must be aware that there is still a residual risk of unwanted pregnancies. It is also possible to keep stallions together if they are of different rank. Nature already provides this model as well [5].

The group size also does not play a role in whether the stallion husbandry is successful or not. However, it is known that the social memory of horses holds about 20 members at most. In other words, horses can easily interact with up to 20 herd members and are able to assign traits and characteristics to them [26,27]. If this size is exceeded, the herd tends to divide, possibly causing space problems. Therefore, group sizes of 10 to 12 horses are ideal [26].

If a horse is unable to meet its natural needs, undesirable behavior is more likely to occur [28,29,30,31,32,33,34,35,36,37,38]. Spending daily free time in a paddock has a positive impact on the horses’ welfare by lessening stereotypic behaviors and increasing oxytocin levels in their blood [39]. This is independent of gender. Irrgang and Gerken [4] and Zilow [6] found in their work that about half of the stallions in solitary confinement showed behavioral problems. Boredom and stress were given as possible causes for this. It is also known from a study by Bachmann [27] that behavioral disorders occur significantly more frequently in solitary confinement. Nevertheless, diseases of the respiratory, digestive, and musculoskeletal systems are more noticeable in individual husbandry [29], which can also be due to a higher stress load, poor stable climate, and lack of exercise. It is, therefore, not surprising that group housing had a particularly favorable effect on the healing of diseases of the respiratory and musculoskeletal systems in a study by Szivacz [9].

In an experiment on the effects of social environment and training on the human–animal relationship, group-housed horses responded clearly better to training than singly housed horses [40]. Additionally, singly housed horses bit the trainer more frequently than did group-housed horses [40]. This also demonstrates the benefits of raising young horses in groups for human’s safety.

Weaknesses of the present work are due to the fact that only one scientifically constructed integration trial of stallions in the off-season was available for review. However, some good-quality studies could be found, which independently came to the same conclusion that keeping stallions in groups is possible in principle. Therefore, these positive tendencies should be scientifically investigated by future work and, thus, be proven. For this purpose, surveys of stallion owners to assess the success of group husbandry could be examined. It is also important to differentiate the effect of different group compositions of stallions (i.e., age, breed, and range) to determine advantages and disadvantages and, thus, develop recommendations for species-appropriate stallion husbandry.

## 5. Conclusions

In conclusion, the keeping of stallions in groups is possible in principle. It only poses a risk if the necessary requirements for this type of husbandry are not taken into account. If the size and design of the exercise area/pasture, the group constellations, and the characters of the stallions are considered, keeping stallions in groups represents the most species-appropriate form of husbandry for them. This takes into account animal welfare aspects and complies best with the requirements of modern horse husbandry. However, the integration of the stallion into an existing group should only be carried out by qualified, experienced horse owners, who must proceed professionally and step by step. Consequently, stress, disadvantages, and the potential for injury can be reduced to a minimum or, ideally, avoided altogether, and the wellbeing and mental and physical health can be supported in the best possible way. Behavioral problems, aggression, and diseases of the respiratory, nutritional, and musculoskeletal systems, on the other hand, are evidence of existing, often serious deficiencies in husbandry management, which result not least from the stress of isolation from individual husbandry.

## Data Availability

The data presented in this study are available on request from the corresponding author.
